# Adolescent exposure to the Spice/K2 cannabinoid JWH-018 impairs sensorimotor gating and alters cortical perineuronal nets in a sex-dependent manner

**DOI:** 10.1038/s41398-023-02469-4

**Published:** 2023-05-25

**Authors:** Cristina Izquierdo-Luengo, Marc Ten-Blanco, María Ponce-Renilla, Ramiro Perezzan, Inmaculada Pereda-Pérez, Fernando Berrendero

**Affiliations:** Instituto de Investigaciones Biosanitarias, Faculty of Experimental Sciences, Universidad Francisco de Vitoria, Madrid, Spain

**Keywords:** Neuroscience, Learning and memory

## Abstract

The consumption of synthetic cannabinoids during adolescence is reported to be a risk factor for the appearance of psychiatric disorders later in life. JWH-018 was identified as one of the primary psychoactive components present in Spice/K2 preparations. This study evaluated the short- and long-term consequences of exposure to JWH-018 during the adolescence in anxiety-like behavior, fear extinction, and sensorimotor gating in male and female mice. Alterations in anxiety varied depending on the time interval between treatment and behavioral analysis along with sex, while no changes were observed in the extinction of fear memory. A decrease in prepulse inhibition of the startle reflex was revealed in male, but not female, mice at short- and long-term. This behavioral disturbance was associated with a reduction in the number of perineuronal nets in the prelimbic and infralimbic regions of the prefrontal cortex in the short-term. Furthermore, adolescent exposure to JWH-018 induced an activation of microglia and astrocytes in the prefrontal cortex of male mice at both time intervals. A transitory decrease in the expression of GAD67 and CB2 cannabinoid receptors in the prefrontal cortex was also found in male mice exposed to JWH-018. These data reveal that the treatment with JWH-018 during the adolescence leads to long-lasting neurobiological changes related to psychotic-like symptoms, which were sex-dependent.

## Introduction

Recreational use of synthetic cannabinoids (SCBs), a novel range of psychoactive substances which have similar effects like ∆^9^-tetrahydrocannabinol (∆^9^-THC), is an increasing public health problem mainly in Western societies [1]. SCBs were promoted by internet retailers and European ‘head shops’ as meditation potpourris and tropical incense products under names such as K2 and Spice [2]. Wrapped in foil packages, these herbal mixtures typically contain a combination of several structural classes of SCBs which have been linked to more adverse health effects than natural cannabinoids [3]. Thus, acute intoxication with SCBs has been related to tachycardia, hypertension, visual and auditory hallucinations, mydriasis, agitation and anxiety, seizures, tachypnea, nausea and vomiting [1]. Most alarming, SCBs abuse in some individuals can result in death [4]. Adolescents [5] and military personnel [6] are the most frequent users probably due to easy accessibility and limited availability of selective and sensitive rapid analytical methods for screening these compounds [7].

The naphthoylindole JWH-018 was identified as one of the primary psychoactive components present in Spice/K2 preparations [8]. Initially developed for therapeutic purposes, JWH-018 is considered the prototypical compound of the so-called “first-generation” class of synthetic cannabinoids. JWH-018 is a potent agonist at cannabinoid type-1 (CB1R) and cannabinoid type-2 (CB2R) receptors, showing approximately a four-fold increased activity at the CB1R and about a ten-fold affinity at the CB2R compared with ∆^9^-THC [9]. In animal models, JWH-018 reproduces the typical “tetrad” effects of THC which are hypothermia, analgesia, hypolocomotion and catalepsy [10], impairs memory retention [11, 12] as well as sensorimotor responses [13, 14], and triggers electrographic seizures [15]_._ Recently, repeated JWH-018 administration was found to induce an anxiety-like phenotype, transitory reductions of sensorimotor gating, and an aversive state upon withdrawal [16]. However, the possible long-lasting behavioral and biochemical changes induced by adolescent JWH-018 exposure are poorly understood.

As previously mentioned, adolescents and young adults show the highest rate of SCBs use [5], which is of particular concern because this period is crucial to generate efficient neuronal pathways by constant neuroplastic shaping, synaptic reorganization and neurochemical changes [17]. Indeed, preclinical studies indicate that cannabinoid exposure during adolescence impacts neurodevelopmental processes and behavior [18], including those normally mediated by the endocannabinoid system [19, 20].

In this study, we investigated the short- and long-term consequences of adolescent exposure to JWH-018 on key neurobehavioral responses associated with SCBs toxicity in humans. Anxiety, fear extinction, and sensorimotor gating were evaluated in male and female mice after treatment with JWH-018 during the adolescence. Possible neurochemical alterations related to these behavioral responses were also studied.

## Materials and methods

### Animals

Adolescent and adult C57BL/6J male and female mice (Charles River) were used in these experiments. Mice were housed 3–4 per cage in a temperature (21 ± 1 °C)—and humidity (55 ± 10%)-controlled room under a 12 h light/dark cycle. All behavioral studies were performed during the light period. Tests were conducted in alternate weeks in male and female mice. Mice were randomly assigned in the different experimental groups. Food and water were available ad libitum. All behavioral data were obtained by experimental observers blinded to the experimental conditions. Experimental procedures were conducted in accordance with the guidelines of the European Communities Directive 2010/63/EU and Spanish Regulations RD 1201/2005 and 53/2013 regulating animal research and approved by the local ethical committee (CEEA-UFV).

### Drugs

JWH-018 (Tocris) was prepared in a 5% ethanol, 5% Tween-80 and 90% saline solution, and was intraperitoneally (i.p.) administered at doses of 0.5, 1 and 1.5 mg/kg (10 ml/kg of body weight). Doses used were based on previous studies [11, 13] in mice.

### Experimental designs

#### JWH-018 treatment during adolescence

**Fig. 1:**
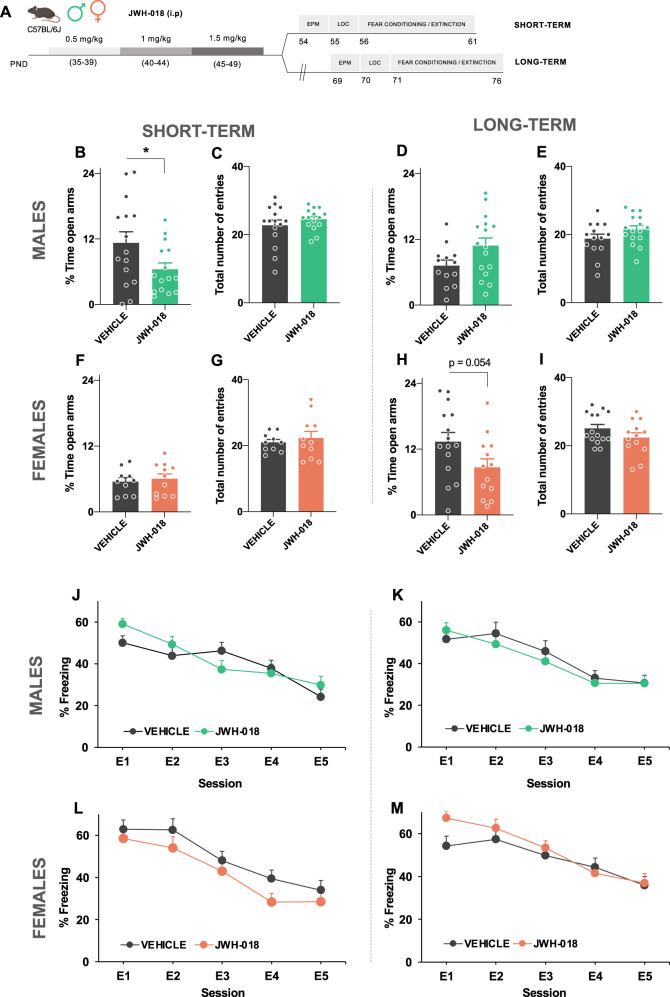
JWH-018 exposure during adolescence alters anxiety-like behavior, but not fear extinction, depending on the sex and the time interval between treatment and behavioral analysis. **A** Schematic representation of the experimental design. **B**–**M** Effects of adolescent exposure to JWH-018 (PND 35–39: 0.5 mg/kg, PND 40–44: 1 mg/kg, and PND 45–49: 1.5 mg/kg) or vehicle in anxiety-like behavior in the EPM **B**–**I** and fear conditioning and extinction **J**–**M** in male mice at short- **B**, **C**, **J** and long-term **D**, **E**, **K**, and female mice at short- **F**, **G**, **L** and long-term **H**, **I**, **M** (*n* = 10–16 mice per group). Percentage of time spent in the open arm and total number of entries are shown for the EPM. Time course of the freezing levels scored during cued fear extinction trials is shown for fear memory processing. Data are expressed as mean ± SEM. *p < 0.05 (comparison between JWH-018 and vehicle; Student’s t-test). PND postnatal day, EPM elevated plus maze, E1-E5 extinction trials.

**Fig. 2:**
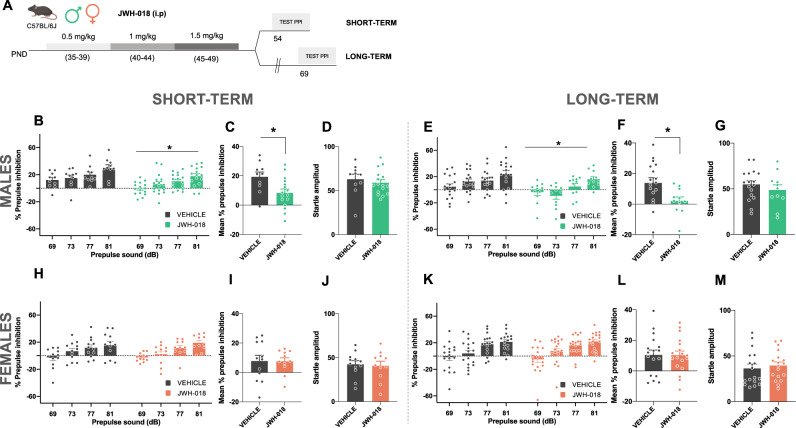
JWH-018 exposure during adolescence impairs sensorimotor gating in male, but not female, mice at short- and long-term. **A** Schematic representation of the experimental design. **B**–**M** Effects of adolescent exposure to JWH-018 (PND 35–39: 0.5 mg/kg, PND 40–44: 1 mg/kg, and PND 45–49: 1.5 mg/kg) or vehicle in sensorimotor gating in male mice at short- **B**–**D** and long-term **E**–**G**, and female mice at short- **H**–**J** and long-term **K**–**M** (*n* = 10–18 mice per group). Percentage of prepulse inhibition, mean of the percentage of prepulse inhibition, and startle response amplitude are shown. Data are expressed as mean ± SEM. *p < 0.05 (comparison between JWH-018 and vehicle group; two-way ANOVA with repeated measures, treatment **B**, **E**; Student’s t-test **C**, **F**). PND postnatal day, dB decibels.

The short- and long-term effects of the exposure to JWH-018 during the adolescence on locomotion, anxiety-like behavior, cued fear conditioning and extinction, and prepulse inhibition (PPI) of the startle reflex were evaluated in both male and female mice. The temporal boundaries of adolescence, considered a vulnerable period to the central effects of drugs [21, 22], are not exactly defined neither in humans nor in rodents [23]. Therefore, based on previous studies [24], mice were treated with increasing doses of JWH-018 (PND 35–39: 0.5 mg/kg, PND 40–44: 1 mg/kg, and PND 45–49: 1.5 mg/kg) or vehicle in order to avoid drug tolerance for 15 days. Short- and long-term effects were analyzed 5 (PND 54) and 20 (PND 69) days respectively after the end of the treatment, as described in Figs. 1A and 2A. The interval of time between adolescent treatment and the different behavioral assays is based on previous reports [24, 25]. Different cohorts of animals were used for the experiments of locomotion, anxiety and fear extinction (males, *n* = 15, short-term, *n* = 14–16, long-term; females, *n* = 10–11, short-term, *n* = 13–15, long-term), and for the experiments of PPI (males, *n* = 10–16, short-term, *n* = 11–17, long-term; females, *n* = 11–12, short-term, *n* = 17–18, long-term). Tissues were obtained 24 h after the PPI test to carry out biochemical experiments in male mice. For short-term, an additional experimental batch was performed to complete the number of mice required. For immunofluorescence experiments, the number of mice was 6–7 (short-term) and 5–7 (long-term). For RT-PCR experiments, the number of mice was 8–9 (short-term) and 6–10 (long-term). The number of animals used in this study is in the usual range of similar experiments previously published. Each experimental sequence was performed once.

#### JWH-018 treatment during adulthood

**Fig. 3:**
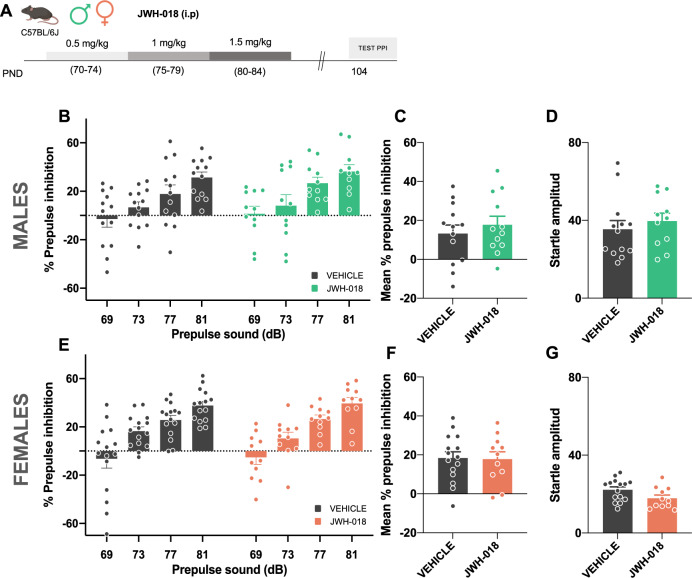
JWH-018 exposure during adulthood does not modify sensorimotor gating in male and female mice. **A** Schematic representation of the experimental design. **B**–**G** Effects of exposure to JWH-018 during adulthood (PND 70–74: 0.5 mg/kg, PND 75–79: 1 mg/kg, and PND 80–84: 1.5 mg/kg) or vehicle in male (**B**–**D**) and female (**E**–**G**) mice (*n* = 11–15 mice per group). Percentage of prepulse inhibition, mean of the percentage of prepulse inhibition, and startle response amplitude are shown. Data are expressed as mean ± SEM. PND postnatal day, dB decibels.

To elucidate whether adolescence is a period of susceptibility to the effects of JWH-018, a similar protocol was performed in adult male and female mice (Fig. 3A). Starting at PND 70, mice were administered with increasing doses of JWH-018 (PND 70–74: 0.5 mg/kg, PND 75–79: 1 mg/kg, and PND 80–84: 1.5 mg/kg). Behavioral evaluation was performed at PND 104 (Fig. 3A), 20 days after the end of the treatment. The number of mice used was 11–13 for males, and 11–15 for females.

### Behavioral experiments

#### Elevated plus maze

Anxiety-like behavior was evaluated using an elevated plus maze (EPM), which consisted in four arms (25 × 5 cm) set in cross from a central square (5 × 5 cm) and raised 30 cm from the ground. Two opposite arms were delimited by vertical walls (closed arms), although the two other arms had unprotected edges (open arms). The apparatus was indirectly illuminated with 40–50 lux. The 5 min performance was recorded through a videocamara located above the maze. Results are expressed as total entries to the open and closed arms, and the percentage of time spent in the open arms with respect to the total amount of time spent in both closed and open arms.

#### Cued fear conditioning and extinction

Experiments were performed as previously reported with slight modifications [24, 26]. Mice were individually placed in the test chamber (LE116, Panlab, Harvard Instruments) made of black methacrylate walls with a transparent front door. The box (25 × 25 x 25 cm) was located inside a soundproof module with a ventilation fan to provide a background noise and attenuate nearby sounds. The chamber floor was formed by parallel stainless-steel bars (2 mm of diameter and 6 mm spaced) connected to a shock generator (LE100-26 module, Panlab, Harvard Instruments). A high-sensitivity weight transducer (load cell unit) was used to record the signal generated by the animal movement intensity. Experimental software PACKWIN V2.0 automatically calculated the percentage of immobility time for each experimental phase. Between each animal trial, the chamber was cleaned with 70% ethanol and then water to avoid olfactory cues. Mice were individually conditioned after a 180 s habituation with 3 cue tones (3 kHz, 80 dB) of 30 s long (10 s interval). Each cue tone (conditioned stimulus, CS) co-terminated with a 0.7 mA foot-shock of 1 s duration (unconditioned stimulus, US). Fear extinction sessions (E1-E5) took place 24, 48, 72, 96 and 120 h after the conditioning day in a novel environment (white walls, transparent cylinder, and smooth floor), and after an acclimatation period, 4 cue tones (CS) were presented with an interval period of 10 s. Freezing behavior, a rodent’s natural response to fear, was automatically evaluated and defined as complete lack of movement, except for breathing for more than 800 ms. Data were expressed as percentage of freezing behavior during the time the sound was active.

#### Prepulse Inhibition of the startle reflex

Prepulse inhibition (PPI) of the startle reflex was tested in two automated StartFear combined system chambers (LE116, Panlab, Harvard Instruments) which were calibrated to ensure equivalent stabilimeter sensitivity. Mice were daily habituated to a non-restrictive Plexiglas cylinder anchored to a high sensitivity transducer for 5 min with background white noise (65 dB) 4 days prior to test. The test started with an acclimatation period of 5 min followed by 5 pulse trials (120 dB, 40 ms) for startle accommodation. The experimental protocol consisted of 10 blocks with 3 or 12 trials each, randomly presented with an inter-trial interval of 10–30 s: no stimulus (12x) (background white noise), pulse alone (12x) (120 dB, 40 ms), pulse preceded by 4 prepulse intensities (12x each) (4, 8, 12 and 16 dB above background noise, 20 ms duration, 100 ms before pulse) and prepulse alone (3x each). Finally, 5 pulse trials were delivered. Initial and final pulses were not included in the final analysis. A background white noise was generated throughout the whole experiment. Startle amplitude was automatically detected by PACKWIN V2.0 software. PPI was calculated as: 100 x (mean startle response – mean prepulse inhibited startle response) / (mean startle response).

#### Locomotion

Changes in locomotor activity were assessed by using locomotor activity boxes (27 × 27 x 21 cm, Cibertec). Mice were individually placed in locomotor cages with low luminosity. Activity was measured as the total number of times the animal crossed an infrared beam during 15 min.

### Tissue preparation for immunofluorescence

Twenty-four h after the PPI test, mice were deeply anesthetized by i.p. injection of ketamine, xylazine and saline solution prior to intracardiac perfusion. Mice were perfused with 1X phosphate buffer saline (PBS) followed by 4% paraformaldehyde. Afterward, brains were post-fixed in 4% paraformaldehyde 24 h and then dehydrated by sequential transfer to 15 and 30% of sucrose in PBS 1X (4 °C). Coronal frozen sections of 20 μm thickness were obtained in a cryostat from 2.10 to 1.54 mm relative to bregma for prefrontal cortex. Brain slices were stored in a cryoprotective solution (20% glycerol, 30% ethylenglycol in PBS 1X) at −20 °C until use. The number of male mice was 6–7 (short-term) and 5–7 (long-term).

### Immunofluorescence

#### Parvalbumin and perineuronal nets

Floating slices were 3 times rinsed in PBS 1X and then treated with blocked solution (4% normal goat serum, 0.1% Triton X-100, 0.1% bovine serum albumin in PBS 1X) for 1.5 h at room temperature. Slices were incubated overnight at 4 °C with the primary antibodies prepared in blocked solution. Rabbit anti-parvalbumin (PV) (1:2000, ab11427, Abcam) and Wisteria floribunda agglutinin combined with fluorescein (1:1000, FL-1351, Vector Laboratories) to label perineuronal nets (PNNs) were used. Next day, after three rinses with PBS 1X (10 min), sections were incubated with the secondary antibody AlexaFluor-594 (1:500, A-11012, Invitrogen) for PV labeling at room temperature for 1 h in blocked solution. Slices were washed 3 times in PBS 1X and mounted with Fluoromount-DAPI (Invitrogen).

#### Iba-1 and GFAP

The same protocol previously described was used, applying the specific antibodies. Primary antibodies used were rabbit anti-Iba-1 (1:1000, 019-19741, Wako) and guinea pig anti-GFAP (1:1000, 173 004, Synaptic system) to label microglial cells and astrocytes, respectively. The secondary antibodies employed were AlexaFluor-594 (1:500, A-11012, Invitrogen) for Iba-1 and AlexaFluor-488 (1:500, A-11073, Invitrogen) for GFAP labeling.

### Immunofluorescence image analysis

#### Parvalbumin and perineuronal nets

Immunostained sections were observed under a Zeiss LSM 900 confocal microscope, using a 20x/0.5 dry objective (Zeiss, CLSM, Germany). Images were acquired through a z-plane (1μm/stack, 8 stacks, 16-bit, 1024 × 1024) and the z-stack was obtained through a maximum projection. A 500 μm squared region of interest (ROI) was delimited for quantification in each infralimbic (IL), prelimbic (PL) and orbitofrontal (OBF) subregions of the prefrontal cortex. The number of positive PV, PNNs and % of PV surrounded by PNNs was semiautomatically detected by using the Pipsqueak tool ® [27] for FIJI (FIJI is just ImageJ) software. For all areas, 5–7 images per animal were quantified.

#### Iba-1 and GFAP

The stained sections were analyzed at 40 × /0.5 objective using a Zeiss LSM 900 confocal microscope (Zeiss, CLSM, Germany). Images were taken through a z-plane (0.5 μm/stack, 10 stacks, 16-bit, 1024 × 1024) and the quantification was carried out through a sum slides projection (32-bit). A quantification ROI of 320 × 320 μm located in the intermediate region between the IL and PL subareas of the prefrontal cortex was chosen. FIJI (FIJI is just Image J) software was used to calculate fluorescence intensity of GFAP stain. The “freehand selection” tool was used to quantify soma area and perimeter of Iba-1-stained cells. Five to seven images per animal were analyzed.

### Quantitative RT-PCR analysis

Prefrontal cortex tissues were extracted 24 h after the PPI test and immediately stored at −80 °C (n = 8–9 (short-term) and n = 6–10 (long-term)). The RNA was purified with the RiboPure™ KIT (Invitrogen) and the reverse transcription was performed with 1μg of total RNA and the SuperScript™ II Reverse Transcriptase (Invitrogen). PCR reactions were conducted using PrimePCR™ Probe Assay (Bio-Rad) to quantify mRNA levels of glutamic acid decarboxylase, 67 kDa isoform (GAD67) (ID: qMmuCEP0060617), brain derived neurotrophic factor (BDNF) (ID: qMmuCEP0058759), synaptophysin (SYP) (ID: qMmuCIP0035577), CB1R (ID: qMmuCEP0038879) and CB2R (ID: qMmuCEP0039299). To evaluate postsynaptic density protein 95 (PSD95) (ID: 4453320), TaqMan™ Gene Expression Assay (Applied Biosystems™) was used. GAPDH (ID: qMmuCEP0039581) expression was used as endogenous control gene for normalization. PCR assays were carried out with the CFX Connect Real-Time PCR Detection System (Bio-Rad). The fold changes in gene expression of JWH-018 treated animals in comparison with controls were calculated using the 2^−ΔΔCt^ method.

### Statistical Analysis

Before the analysis, all data were checked for normality (Kolmogorov-Smirnov test) and homogeneity of variances (Bartlett’s test). Statistical analysis was carried out using unpaired Student t-test (with Welch’s correction when appropriate), two-way ANOVA of repeated measures followed by Newman-Keuls post hoc comparisons after significant interactions between factors. When parametric normality test was violated, a Mann-Whitney nonparametric test was used. Pearson’s correlation coefficient was used to analyze the relationship between two variables. Outliers were excluded if they were >2 standard deviations from the mean. All data are expressed as mean ± SEM. A p value < 0.05 was used to determine statistical significance. The statistical analysis was performed using STATISTICA (StatSoft) software and GraphPad Prism 9.

## Results

### Short- and long-term consequences on anxiety and fear extinction in adolescent mice exposed to JWH-018

Adolescent male and female mice were treated with increasing doses of JWH-018 during 15 days (PND 35–39: 0.5 mg/kg, PND 40–44: 1 mg/kg, and PND 45–49: 1.5 mg/kg) (Fig. 1A). Body weight was daily evaluated along JWH-018 treatment. The weight gain of mice treated with JWH-018 was lower than those exposed to vehicle in both sexes (Supplementary Fig. 1) (treatment effect: F_1,27_ = 4.88, p < 0.05 and F_1,22_ = 5.12, p < 0.05, for male and female mice, respectively), in agreement with previous reports evaluating effects of adolescent THC exposure [24, 28]. Locomotor activity, anxiety-like behavior and fear memory processing were analyzed 5 (short-term) or 20 (long-term) days after the finishing of JWH-018 treatment (Fig. 1A). No changes in locomotion were observed in either males or females (Supplementary Fig. 2). By using the EPM, JWH-018 induced an anxiogenic-like effect in males in the short-term (p < 0.05) (Fig. 1B). This effect was specific to the early period as they recovered when they reached the adulthood (Fig. 1D). In contrast, no differences in anxiety were observed in female mice in the short-term (Fig. 1F), while there was a clear long-term anxiogenic trend (p = 0.054) (Fig. 1H). Total number of entries were not modified in either males or females (Fig. 1C, E, G, I). Aversive memory processing was evaluated by a cued fear conditioning paradigm. The administration of JWH-018 did not alter cued fear extinction in both males (Fig. 1J, K) and females (Fig. 1L, M) in the short- and the long-term. These results suggest the existence of sex-specific effects in unconditioned anxiety due to JWH-018 exposure during the adolescence.

### Short- and long-term consequences on sensorimotor gating in adolescent mice exposed to JWH-018

Impairments of PPI of the startle reflex, a sensorimotor gating process, are observed in patients with schizophrenia [29] and is considered a marker of psychotic-like behavior [30]. By using the same experimental protocol of JWH-018 administration previously described (Fig. 2A), we studied possible PPI alterations in both male and female mice. Interestingly, a significant decrease of PPI of the startle reflex was revealed in male mice in both short- (treatment effect: F_1,24_ = 6.79, p < 0.05) (Fig. 2B) and long-term (treatment effect: F_1,26_ = 6.06, p < 0.05) (Fig. 2E). An overall reduction of PPI due to JWH-018 exposure was observed when representing mean PPI score at both time periods (p < 0.05) (Fig. 2C, F). This effect was independent of baseline changes in startle amplitude (Fig. 2D, G), ruling out an impact of startle reaction in the PPI modifications observed. In contrast to male mice, adolescent exposure to JWH-018 did not modify PPI of the startle reflex in females (Fig. 2H, I, K, L). The magnitude of startle reflex was also not altered by JWH-018 injection (Fig. 2J, M) in these mice. These results suggest a sex-dependent alteration on sensorimotor gating due to the treatment with the synthetic cannabinoid JWH-018.

To elucidate whether immature brain represents a period of development more susceptible to the effects of JWH-018, we evaluated the consequences of the synthetic cannabinoid exposure directly on adult animals (Fig. 3A). Possible changes in sensorimotor gating were assessed 20 days following the last day of JWH-018 administration (Fig. 3A), as previously studied after treatment during the adolescent period. Notably, no differences in PPI were observed between vehicle and JWH-018 groups in male mice (Fig. 3B–D), indicating that adolescence represents a sensitive window for the harmful consequences of JWH-018 exposure. Moreover, JWH-018 administration in adult females did not modify PPI (Fig. 3E–G), in agreement with the lack of effect previously observed in adolescent female mice treated with this synthetic cannabinoid.

### Short- and long-term consequences on the density of cortical parvalbumin-expressing interneurons and perineuronal nets in adolescent mice exposed to JWH-018

**Fig. 4:**
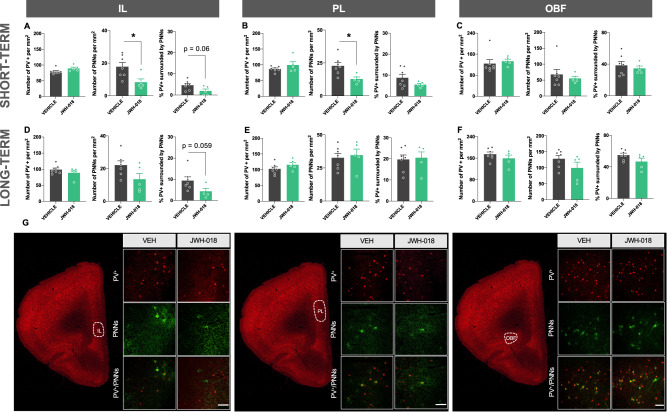
JWH-018 exposure during adolescence alters perineuronal nets density in cortical subregions in male mice. **A**–**F** Number of PV+, PNNs and PV+ surrounded by PNNs in the IL (**A**, **D**), PL (**B**, **E**) and OBF (**C**, **F**) in male mice exposed to JWH-018 during adolescence at short- (**A**–**C**) and long-term (**D**–**F**). Tissue was obtained 24 h after the prepulse inhibition test (*n* = 5–7 mice per group). **G** Representative images of each cortical subregion obtained by fluorescence microscopy labelling PV+ (red) and PNNs (green) of short-term experiments. Scale bar represents 100 µm. Data are expressed as mean ± SEM. *p < 0.05 (comparison between JWH-018 and vehicle; Student’s t-test). IL infralimbic prefrontal cortex, PL prelimbic prefrontal cortex, OBF orbitofrontal cortex, PV+  positive parvalbumin neuron, PNNs perineuronal nets.

The prefrontal cortex is a brain area directly related to the modulation of sensorimotor gating [31]. Moreover, studies on both patients and animal models of schizophrenia have found alterations in the subpopulation of cortical GABAergic interneurons expressing PV [32, 33]. During development the connectivity and maturation of these interneurons are regulated by the presence of PNNs, specialized regions of the extracellular matrix, which are frequently surrounding PV-expressing neurons [34]. Interestingly, some studies have revealed that patients with schizophrenia show a reduced density of PNNs in the prefrontal cortex [35, 36]. We next studied possible alterations in the density of cortical interneurons expressing PV and PNNs in adolescent male mice exposed to JWH-018, given the deficits previously observed in PPI of the startle reflex in these animals. Notably, the density of PNNs significantly decreased in the IL (Fig. 4A, G) and PL (Fig. 4B, G) (p < 0.05), but not in the OBF (Fig. 4C, G), in male mice 5 days after the end of the treatment with JWH-018. These changes were reversible as no long-term differences were found between groups (Fig. 4D–F). In agreement, the density of PNNs was similar in adult females exposed to JWH-018 during adolescence (Supplementary Fig. 3). The number of neurons expressing PV was not altered by JWH-018 exposure in both males (Fig. 4A–G) and females (Supplementary Fig. 3) in any of the brain regions of the prefrontal cortex analyzed. We also found an almost significant decrease in the percentage of PV neurons surrounded by PNNs in the IL of male, but not in female (Supplementary Fig. 3), mice in both the short- (p = 0.06) and long-term (p = 0.059) (Fig. 4A, D, G).

### Short- and long-term consequences on the microglia morphology and GFAP immunoreactivity in astrocytes in adolescent mice exposed to JWH-018

**Fig. 5:**
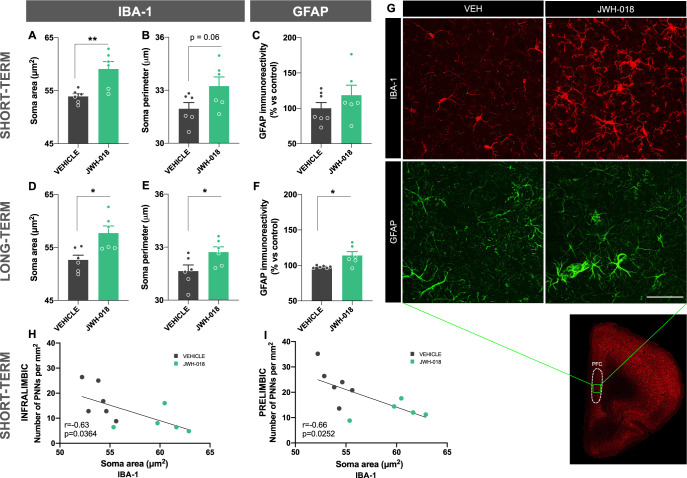
JWH-018 exposure during adolescence induces microglia activation in the prefrontal cortex in male mice. **A**, **B**, **D**, **E** Area and perimeter of microglia soma of Iba-1-stained cells, and **C**, **F** GFAP immunoreactivity in the prefrontal cortex in male mice exposed to JWH-018 during adolescence at short- (**A**–**C**) and long-term (**D**–**F**). Tissue was obtained 24 h after the prepulse inhibition test (*n* = 6 mice per group). **G** Representative images of adult males obtained by fluorescence microscopy labelling Iba-1 (red) and GFAP (green). Scale bar represents 50 µm. **H**, **I** Correlations between the soma area of Iba-1-stained cells and the number of PNNs in the IL (**H**) and PL (**I**) 24 h after the prepulse inhibition test in male mice at short-term (Pearson’s correlation coefficient). Data are expressed as mean ± SEM. *p < 0.05; **p < 0.01 (comparison between JWH-018 and vehicle; Student’s t-test). Iba-1 ionized calcium-binding adapter molecule 1, GFAP glial fibrillary acidic protein, IL infralimbic prefrontal cortex, PL prelimbic prefrontal cortex, PNNs perineuronal nets.

Microglia are a key defense mechanism within the brain, but their activation can result in damage to PNNs through either the release of proteolytic enzymes such as matrix metalloproteinases [37] or directly by stripping PNNs from the neuronal surface [38]. In addition, astrocytes release an array of diverse matrix-remodeling proteases and their inhibitors to tightly control the structural integrity of PNNs [37]. Interestingly, an enhancement of the area of the microglia soma was observed in the prefrontal cortex of male mice exposed to JWH-018 during the adolescent period in both short (p < 0.01) (Fig. 5A, G) and long-term (p < 0.05) (Fig. 5D, G). Accordingly, the perimeter of microglia soma showed a strong trend to increase in the short term (p = 0.06) (Fig. 5B, G) while this enhancement was significant in the long-term (p < 0.05) (Fig. 5E, G) in these animals. No changes were observed in the total number of Iba-1 positive cells (Supplementary Fig. 4). These results indicate that JWH-018 treatment induces a microglia morphology shift to a reactive state characterized by larger amoeboid soma [39]. However, no alterations were revealed in both area and perimeter of the microglia soma in females (Supplementary Fig. 3). A significant negative correlation between microglial activation (soma area) and PNNs density in the IL (p < 0.05) (Fig. 5H) and PL (p < 0.05) (Fig. 5I) was found in the short-term in male mice exposed to JWH-018. All together, these results suggest that exposure to this synthetic cannabinoid in adolescent male mice involves changes in microglial reactivity in the prefrontal cortex which are associated with a decrease of the density of PNNs. These histopathological alterations could contribute to the PPI deficits present in these mice. Indeed, a significant correlation between the percentage of PPI, when representing the prepulses of 69 and 73 dB, and the density of PNNs was found in the IL in the short- and the long-term (p < 0.05) (Supplementary Fig. 5) in male mice.

JWH-018 exposure in male, but not in female (Supplementary Fig. 3), mice enhanced GFAP immunoreactivity in the prefrontal cortex in the long-term (p < 0.05) (Fig. 5F, G). No modifications were observed in the short-term in these mice (Fig. 5C).

### Short- and long-term consequences on the expression of GAD67, SYN, PSD95, BDNF, CB1R and CB2R in adolescent mice exposed to JWH-018

**Fig. 6:**
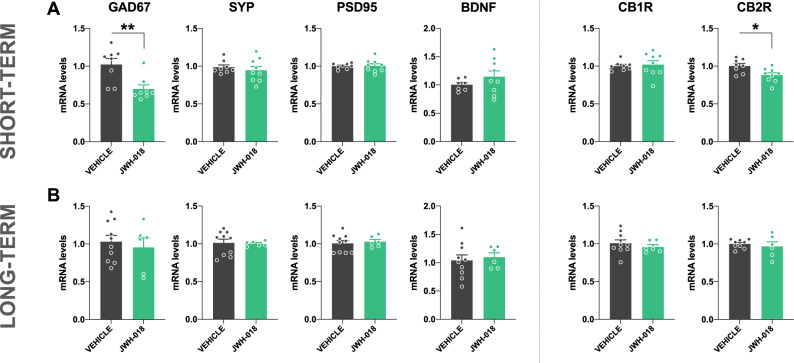
JWH-018 exposure during adolescence induces a decrease in the expression of GAD67 and CB2R in the prefrontal cortex in male mice. **A**, **B** mRNA levels of GAD67, SYP, PSD95, BDNF, CB1R and CB2R in the prefrontal cortex in male mice exposed to JWH-018 during adolescence at short- **A** and long-term **B**. Tissue was obtained 24 h after the prepulse inhibition test (*n* = 6–10 mice per group). Data are expressed as mean ± SEM. *p < 0.05; **p < 0.01 (comparison between JWH-018 and vehicle; Mann-Whitney test for GAD67 expression, and Student’s t-test for CB2R expression). GAD67 glutamic acid decarboxylase 67, SYP synaptophysin, PSD95 postsynaptic density protein 95, BDNF brain derived neurotrophic factor, CB1R cannabinoid receptor type 1, CB2R cannabinoid receptor type 2.

We finally explored whether the expression of molecules related to inhibitory neurotransmission and plasticity could be altered in the short- and long-term in male mice exposed to the synthetic cannabinoid JWH-018 during the adolescence. Possible changes in the expression of CB1R and CB2R were also investigated. A significant decrease was found in the mRNA levels of GAD67 (p < 0.01) and CB2R (p < 0.05) in the prefrontal cortex in the short-term (Fig. 6A). These alterations were specific to the early period after JWH-018 treatment (Fig. 6B). No differences in the expression of SYN, PSD95, BDNF and CB1R were observed in the short- and the long-term (Fig. 6A, B) in these mice.

## Discussion

Our data show that exposure to the Spice/K2 cannabinoid JWH-018 in adolescent mice induces long-term behavioral consequences which were sex-dependent. Notably, JWH-018 treatment during adolescence, but not in adulthood, triggered impairments of PPI of the startle reflex in young and adult male mice. These alterations were associated with changes in the density of PNNs and microglia morphology in the prefrontal cortex.

Adverse outcomes associated with the consumption of SCBs are distinct from, and markedly more toxic than, those produced by marijuana [3]. Anxiety, panic attacks, and psychotic symptoms are among the most common psychiatric detrimental effects reported from several clinical case studies [3]. However, there is little information about the behavioral and neurochemical consequences of adolescent exposure to these drugs. In the present study, we reported that administration of JWH-018 during adolescence modified anxiety-like behavior in a sex-specific manner. An anxiogenic phenotype was revealed in male, but not in female, mice five days after the JWH-018 administration. In contrast, an anxiogenic trend were observed in females, but not in males, when the animals reached the adulthood. By using the EPM, most of the studies have not shown differences in anxiety in adult male [24, 40, 41] or female [40, 41] rodents due to adolescent ∆^9^-THC exposure, although an anxiogenic-like effect was observed in adult male mice in another report [42]. A recent study revealed an increase of repetitive/compulsive behaviors at adulthood in adolescent male mice exposed to JWH-018, as shown in the nestlet shredding and marble burying tests [43]. Although marble burying test can be used to assess anxiety-like behavior, the heightened marble burying activity of JWH-018 treated male mice could reflect compulsive-like rather than anxiety-like states considering that in a previous study adult male rats showed increased burying scores, but not anxiety alterations in the EPM test, 7 days after JWH-018 discontinuation [16]. Moreover, adolescent exposure to the recently detected synthetic cannabinoid 5F-MDMB-PICA displayed an anxiety-like and compulsive-like state in adult male mice [44]. On the other hand, anxiety disorders characterized by pathological fear, such as post-traumatic stress disorder and phobias, are associated with extinction deficits of aversive memories. Under our experimental conditions, cued fear extinction was not affected in either male or female mice by adolescent exposure to JWH-018. Consistent with this result, the administration of ∆^9^-THC during adolescence did not alter fear extinction in male and female adult mice [24], although impairment of this response was revealed when THC was combined with stress exposure [24].

A critical finding of our study was that adolescent JWH-018 exposure induces a reduction of PPI of the startle reflex at short- and long-term in male, but not in female, mice. Notably, the effect of JWH-018 was age-dependent as revealed by the lack of alterations in the PPI test when the same drug administration regimen was performed directly in adulthood. PPI is a classic preclinical model of sensorimotor gating, with high translational validity between humans and rodents [45], that measures the ability to filter out insignificant sensory information, a cognitive abnormality also seen in schizophrenia [46]. Previous studies evaluating the effects of ∆^9^-THC during adolescence on PPI in rodents have yielded mixed results. Thus, ∆^9^-THC administration induced persistent PPI deficits in adult male rats [47, 48] while an increase in PPI was observed in a recent report [49] in male mice. The consequences of adolescent exposure to JWH-018 on PPI in adulthood were not previously assessed although, in agreement with our study, chronic administration of this synthetic cannabinoid in adult male rats did not induce changes in sensorimotor gating 1 or 7 days after the end of the pharmacological treatment [16]. Interestingly, we found that the effects of adolescent exposure to JWH-018 on PPI responses were modulated by sex, being males more vulnerable than females. Although sex differences are a known and important part of mental illness, they are often overlooked in animal models. Preclinical and clinical studies show differences between sexes in the therapeutic potential and abuse liability of cannabis and cannabinoids [50]. Moreover, sex has been described as a major factor modulating pharmacokinetic, behavioral, and brain activity effects of ∆^9^-THC in adolescent rats [51]. On the other hand, sex differences in CB1R expression have been found between male and female rodents throughout the brain. In this sense, CB1R protein or mRNA was found to be greater in male versus female rats in the prefrontal cortex [52]. In any case, future experiments will be necessary to determine the factors related to this sex-dependent effect including puberty timing, different biochemical responses, tolerance from repeated injections, and hormonal status (i.e., estrous cycle phase) [51]_._

PNNs, condensed aggregates of the extracellular matrix, are important players in the regulation of the maturation and plasticity of interneurons, especially fast-spiking GABAergic interneurons expressing PV [53]. Although the functions of PNNs are not clear yet, they have been involved in the modulation of different cognitive processes and could be considered as a future potential therapeutic target for the treatment of diseases characterized by maladaptive memories [54]. Interestingly, PNNs in the prefrontal cortex are dysregulated in patients with schizophrenia [34, 35], and in animal models that mimic this disorder [55, 56]. Indeed, a link between these specialized regions of the extracellular matrix and the presence of psychosis has been suggested [34]. We observed a reduction in the density of PNNs in the IL and the PL at short-term due to the adolescent exposure to JWH-018 in male mice. Moreover, the percentage of PV neurons surrounded by PNNs in the IL tended to decrease even in the long-term. These alterations could be related to the PPI disruptions present in these animals since a correlation between the density of PNNs and the percentage of PPI was found in the IL. In contrast to the effects of JWH-018, ∆^9^-THC administration during the adolescence did not modify the density of PNNs in the prefrontal cortex in adult male mice [49]. This discrepancy could be due to the different time interval between treatment and testing (shorter in our study), and the higher potency of JWH-018 compared to THC.

Glial cells are among the major extrinsic factors that facilitate the remodeling of PNNs, thereby acting as key regulators of their diverse functions in health and disease [37, 57]. PNNs abundance is dramatically upregulated throughout the healthy adult brain following microglial depletion in mice [58]. Interestingly, PNNs deficits/decreases have been observed across diverse disorders, generally associated with microglial activation [57]. In addition, reactive astrocytes also play a role in PNN disruption after brain injury and trauma [37]. Notably, adolescent JWH-018 exposure induced an activation of microglia and astrocytes in the prefrontal cortex of adult male mice. In agreement, a recent study showed a long-term microglia activation in several brain areas such as the nucleus accumbens and caudate-putamen following adolescent self-administration of JWH-018 [43]. Taken together, it is tempting to hypothesize that exposure to JWH-018 to adolescent mice induces changes in glial cells reactivity and PNNs density in the prefrontal cortex, leading to the appearance of sensorimotor gating deficits in adulthood.

Several studies have shown reductions in the expression of GAD67 in the prefrontal cortex of schizophrenia patients [59, 60], which contribute to dysfunction of this brain region. Simultaneous reductions in the expression of GABAergic markers in the prefrontal cortex and in PPI have been also reported [61]. Notably, adolescent exposure to JWH-018 reduced the expression of GAD67 in the prefrontal cortex in the short term, although normal levels were found in adult male mice. In this sense, lower levels of GAD67 in the prefrontal cortex were shown after ∆^9^-THC administration during adolescence, which resulted in a psychotic-like phenotype in adult female rats [62]. Furthermore, a transitory decrease in the expression of CB2R, but not CB1R, was observed due to adolescent JWH-018 treatment. Several studies present evidence of the contribution of CB2R to the modulation of sensorimotor gating. Interestingly, an increased expression of CB2R in the prefrontal cortex has been recently related to enhanced PPI of the startle reflex in male 129S1/SvImJ mice [63]. Accordingly, CB2R knockout mice showed disrupted PPI at different prepulse intensities [64], while MK801-induced decrease in PPI was attenuated by the CB2R agonists JWH015 [65] and HU-910 [66]. On the other hand, 5-HT_2c_ receptors could also be involved in the PPI deficits caused by adolescent JWH-018 exposure. Indeed, the activation of this serotonin receptor has been previously shown to reverse PPI deficits induced by apomorphine in rats [67], and by the NMDA antagonist MK801 in mice [68]. Moreover, we observed a decrease in the mRNA levels of 5-HT_2c_ receptor in the prefrontal cortex of adolescent male mice exposed to JWH-018 in the short-term (data not shown), a mechanism that will be worth exploring in the future.

In summary, our data show important lasting behavioral and neurobiological changes associated with JWH-018 treatment during adolescence. This study has profound clinical and public health policy implications in terms of limiting adolescents to cannabinoid synthetic compounds exposure that may be particularly neurotoxic during certain neurodevelopmental windows.

## Supplementary Information

Supplemental Material

## References

[1] Davidson C, Opacka-Juffry J, Arevalo-Martin A, Garcia-Ovejero D, Molina-Holgado E, Molina-Holgado F (2017). Spicing up pharmacology: a review of synthetic cannabinoids from structure to adverse events. Adv Pharm.

[2] Castaneto MS, Gorelick DA, Desrosiers NA, Hartman RL, Pirard S, Huestis MA (2014). Synthetic cannabinoids: epidemiology, pharmacodynamics, and clinical implications. Drug Alcohol Depend.

[3] Ford BM, Tai S, Fantegrossi WE, Prather PL (2017). Synthetic pot: not your grandfather’s marijuana. Trends Pharm Sci.

[4] Trecki J, Gerona RR, Schwartz MD (2015). Synthetic cannabinoid–related illnesses and deaths. N. Engl J Med.

[5] Mathews EM, Jeffries E, Hsieh C, Jones G, Buckner JD (2019). Synthetic cannabinoid use among college students. Addictive Behav.

[6] Santangelo O, Baldwin JM, Stogner J (2022). Does cannabis testing in the military drive synthetic cannabinoid use? Self-reported use motivations among justice-involved veterans. Int J Drug Policy.

[7] Bukke VN, Archana M, Villani R, Serviddio G, Cassano T (2021). Pharmacological and toxicological effects of phytocannabinoids and recreational synthetic cannabinoids: Increasing risk of public health. Pharmaceuticals.

[8] Gurney SMR, Scott KS, Kacinko SL, Presley BC, Logan BK (2014). Pharmacology, toxicology, and adverse effects of synthetic cannabinoid drugs. Forensic Sci Rev.

[9] Auwärter V, Dresen S, Weinmann W, Müller M, Pütz M, Ferreirós N (2009). ‘Spice’ and other herbal blends: harmless incense or cannabinoid designer drugs?. J Mass Spectrom.

[10] Marshell R, Kearney-Ramos T, Brents LK, Hyatt WS, Tai S, Prather PL (2014). In vivo effects of synthetic cannabinoids JWH-018 and JWH-073 and phytocannabinoid Δ9-THC in mice: Inhalation versus intraperitoneal injection. Pharm Biochem Behav.

[11] Barbieri M, Ossato A, Canazza I, Trapella C, Borelli AC, Beggiato S (2016). Synthetic cannabinoid JWH-018 and its halogenated derivatives JWH-018-Cl and JWH-018-Br impair novel object recognition in mice: behavioral, electrophysiological and neurochemical evidence. Neuropharmacology.

[12] Li RS, Fukumori R, Takeda T, Song Y, Morimoto S, Kikura-Hanajiri R (2019). Elevation of endocannabinoids in the brain by synthetic cannabinoid JWH-018: mechanism and effect on learning and memory. Sci Rep..

[13] Ossato A, Vigolo A, Trapella C, Seri C, Rimondo C, Serpelloni G (2015). JWH-018 impairs sensorimotor functions in mice. Neuroscience.

[14] Bilel S, Tirri M, Arfè R, Ossato A, Trapella C, Serpelloni G (2020). Novel halogenated synthetic cannabinoids impair sensorimotor functions in mice. Neurotoxicology.

[15] Malyshevskaya O, Aritake K, Kaushik MK, Uchiyama N, Cherasse Y, Kikura-Hanajiri R (2017). Natural (Δ9-THC) and synthetic (JWH-018) cannabinoids induce seizures by acting through the cannabinoid CB1 receptor. Sci Rep..

[16] Pintori N, Castelli MP, Miliano C, Simola N, Fadda P, Fattore L (2021). Repeated exposure to JWH-018 induces adaptive changes in the mesolimbic and mesocortical dopaminergic pathways, glial cells alterations, and behavioural correlates. Br J Pharm.

[17] Sturman DA, Moghaddam B (2011). The neurobiology of adolescence: changes in brain architecture, functional dynamics, and behavioral tendencies. Neurosci Biobehav Rev.

[18] Scheyer AF, Laviolette SR, Pelissier AL, Manzoni OJJ. Cannabis in adolescence: lasting cognitive alterations and underlying mechanisms. Cannabis Cannabinoid Res. 2022. 10.1089/can.2022.0183 10.1089/can.2022.0183 PMC9940816 36301550

[19] Fernández-Ruiz J, Berrendero F, Hernández ML, Ramos JA (2000). The endogenous cannabinoid system and brain development. Trends Neurosci.

[20] Harkany T, Guzmán M, Galve-Roperh I, Berghuis P, Devi LA, Mackie K (2007). The emerging functions of endocannabinoid signaling during CNS development. Trends Pharm Sci.

[21] Schneider M (2013). Adolescence as a vulnerable period to alter rodent behavior. Cell Tissue Res.

[22] Rubino T, Parolaro D (2016). The impact of exposure to cannabinoids in adolescence: Insights from animal models. Biol Psychiatry.

[23] Brust V, Schindler PM, Lewejohann L (2015). Lifetime development of behavioural phenotype in the house mouse (Mus musculus). Front Zool.

[24] Saravia R, Ten-Blanco M, Julià-Hernández M, Gagliano H, Andero R, Armario A (2019). Concomitant THC and stress adolescent exposure induces impaired fear extinction and related neurobiological changes in adulthood. Neuropharmacology.

[25] Ibarra-Lecue I, Mollinedo-Gajate I, Meana JJ, Callado LF, Diez-Alarcia R, Urigüen L (2018). Chronic cannabis promotes pro-hallucinogenic signaling of 5-HT2A receptors through Akt/mTOR pathway. Neuropsychopharmacology.

[26] Flores Á, Valls-Comamala V, Costa G, Saravia R, Maldonado R, Berrendero F (2014). The hypocretin/orexin system mediates the extinction of fear memories. Neuropsychopharmacology.

[27] Slaker ML, Harkness JH, Sorg BA (2016). A standardized and automated method of perineuronal net analysis using Wisteria floribunda agglutinin staining intensity. IBRO Rep..

[28] Scherma M, Dessì C, Muntoni AL, Lecca S, Satta V, Luchicchi A (2016). Adolescent Δ9-tetrahydrocannabinol exposure alters WIN55,212-2 self-administration in adult rats. Neuropsychopharmacology.

[29] Mena A, Ruiz-Salas JC, Puentes A, Dorado I, Ruiz-Veguilla M, de la Casa LG (2016). Reduced prepulse inhibition as a biomarker of schizophrenia. Front Behav Neurosci.

[30] Carceles-Cordon M, Mannara F, Aguilar E, Castellanos A, Planagumà J, Dalmau J (2020). NMDAR antibodies alter dopamine receptors and cause psychotic behavior in mice. Ann Neurol.

[31] Tóth A, Petykó Z, Gálosi R, Szabó I, Karádi K, Feldmann Á (2017). Neuronal coding of auditory sensorimotor gating in medial prefrontal cortex. Behav Brain Res.

[32] Lodge DJ, Behrens MM, Grace AA (2009). A loss of parvalbumin-containing interneurons is associated with diminished oscillatory activity in an animal model of schizophrenia. J Neurosci.

[33] Gonzalez-Burgos G, Cho RY, Lewis DA (2015). Alterations in cortical network oscillations and parvalbumin neurons in schizophrenia. Biol Psychiatry.

[34] Alcaide J, Guirado R, Crespo C, Blasco-Ibáñez JM, Varea E, Sanjuan J (2019). Alterations of perineuronal nets in the dorsolateral prefrontal cortex of neuropsychiatric patients. Int J Bipolar Disord.

[35] Mauney SA, Athanas KM, Pantazopoulos H, Shaskan N, Passeri E, Berretta S (2013). Developmental pattern of perineuronal nets in the human prefrontal cortex and their deficit in schizophrenia. Biol Psychiatry.

[36] Enwright JF, Sanapala S, Foglio A, Berry R, Fish KN, Lewis DA (2016). Reduced labeling of parvalbumin neurons and perineuronal nets in the dorsolateral prefrontal cortex of subjects with schizophrenia. Neuropsychopharmacology.

[37] Tewari BP, Chaunsali L, Prim CE, Sontheimer H (2022). A glial perspective on the extracellular matrix and perineuronal net remodeling in the central nervous system. Front Cell Neurosci.

[38] Crapser JD, Spangenberg EE, Barahona RA, Arreola MA, Hohsfield LA, Green KN (2020). Microglia facilitate loss of perineuronal nets in the Alzheimer’s disease brain. EBioMedicine.

[39] Kohman RA, Rhodes JS (2013). Neurogenesis, inflammation and behavior. Brain Behav Immun.

[40] Rubino T, Realini N, Castiglioni C, Guidali C, Viganó D, Marras E (2008). Role in anxiety behavior of the endocannabinoid system in the prefrontal cortex. Cereb Cortex.

[41] Zuo Y, Iemolo A, Montilla-Perez P, Li HR, Yang X, Telese F (2022). Chronic adolescent exposure to cannabis in mice leads to sex-biased changes in gene expression networks across brain regions. Neuropsychopharmacology.

[42] Murphy M, Mills S, Winstone J, Leishman E, Wager-Miller J, Bradshaw H (2017). Chronic adolescent Δ9-tetrahydrocannabinol treatment of male mice leads to long-term cognitive and behavioral dysfunction, which are prevented by concurrent cannabidiol treatment. Cannabis Cannabinoid Res.

[43] Margiani G, Castelli MP, Pintori N, Frau R, Ennas MG, Orrù V (2022). Adolescent self-administration of the synthetic cannabinoid receptor agonist JWH-018 induces neurobiological and behavioral alterations in adult male mice. Psychopharmacol (Berl).

[44] Musa A, Simola N, Piras G, Caria F, Onaivi ES, De Luca MA (2020). Neurochemical and behavioral characterization after acute and repeated exposure to novel synthetic cannabinoid agonist 5-MDMB-PICA. Brain Sci.

[45] van den Buuse M (2010). Modeling the positive symptoms of schizophrenia in genetically modified mice: pharmacology and methodology aspects. Schizophr Bull.

[46] Renard J, Vitalis T, Rame M, Krebs MO, Lenkei Z, le Pen G (2016). Chronic cannabinoid exposure during adolescence leads to long-term structural and functional changes in the prefrontal cortex. Eur Neuropsychopharmacol.

[47] Renard J, Rosen LG, Loureiro M, de Oliveira C, Schmid S, Rushlow WJ (2017). Adolescent cannabinoid exposure induces a persistent sub-cortical hyper-dopaminergic state and associated molecular adaptations in the prefrontal cortex. Cereb Cortex.

[48] Abela AR, Rahbarnia A, Wood S, Lê AD, Fletcher PJ (2019). Adolescent exposure to Δ9-tetrahydrocannabinol delays acquisition of paired-associates learning in adulthood. Psychopharmacol (Berl).

[49] Garcia-Mompo C, Curto Y, Carceller H, Gilabert-Juan J, Rodriguez-Flores E, Guirado R (2020). Δ-9-Tetrahydrocannabinol treatment during adolescence and alterations in the inhibitory networks of the adult prefrontal cortex in mice subjected to perinatal NMDA receptor antagonist injection and to postweaning social isolation. Transl Psychiatry.

[50] Cooper ZD, Craft RM (2018). Sex-dependent effects of cannabis and cannabinoids: a translational perspective. Neuropsychopharmacology.

[51] Ruiz CM, Torrens A, Castillo E, Perrone CR, Cevallos J, Inshishian VC (2021). Pharmacokinetic, behavioral, and brain activity effects of ∆9-tetrahydrocannabinol in adolescent male and female rats. Neuropsychopharmacology.

[52] Castelli M, Fadda P, Casu A, Spano M, Casti A, Fratta W (2014). Male and female rats differ in brain cannabinoid CB1 receptor density and function and in behavioural traits predisposing to drug addiction: effect of ovarian hormones. Curr Pharm Des.

[53] Wingert JC, Sorg BA (2021). Impact of Perineuronal Nets on Electrophysiology of Parvalbumin Interneurons, Principal Neurons, and Brain Oscillations: A Review. Front Synaptic Neurosci.

[54] Reichelt AC, Hare DJ, Bussey TJ, Saksida LM (2019). Perineuronal nets: plasticity, protection, and therapeutic potential. Trends Neurosci.

[55] Paylor JW, Lins BR, Greba Q, Moen N, de Moraes RS, Howland JG (2016). Developmental disruption of perineuronal nets in the medial prefrontal cortex after maternal immune activation. Sci Rep..

[56] Matuszko G, Curreli S, Kaushik R, Becker A, Dityatev A (2017). Extracellular matrix alterations in the ketamine model of schizophrenia. Neuroscience.

[57] Crapser JD, Arreola MA, Tsourmas KI, Green KN (2021). Microglia as hackers of the matrix: sculpting synapses and the extracellular space. Cell Mol Immunol.

[58] Liu YJ, Spangenberg EE, Tang B, Holmes TC, Green KN, Xu X (2021). Microglia elimination increases neural circuit connectivity and activity in adult mouse cortex. J Neurosci.

[59] Hashimoto T, Volk DW, Eggan SM, Mirnics K, Pierri JN, Sun Z (2003). Gene expression deficits in a subclass of GABA neurons in the prefrontal cortex of subjects with schizophrenia. J Neurosci.

[60] Curley AA, Arion D, Volk DW, Asafu-Adjei JK, Sampson AR, Fish KN (2011). Cortical deficits of glutamic acid decarboxylase 67 expression in schizophrenia: Clinical, protein, and cell type-specific features. Am J Psychiatry.

[61] Toriumi K, Oki M, Muto E, Tanaka J, Mouri A, Mamiya T (2016). Prenatal phencyclidine treatment induces behavioral deficits through impairment of GABAergic interneurons in the prefrontal cortex. Psychopharmacol (Berl).

[62] Zamberletti E, Beggiato S, Steardo L, Prini P, Antonelli T, Ferraro L (2014). Alterations of prefrontal cortex GABAergic transmission in the complex psychotic-like phenotype induced by adolescent delta-9-tetrahydrocannabinol exposure in rats. Neurobiol Dis.

[63] Ten-Blanco M, Pereda-Pérez I, Izquierdo-Luengo C, Berrendero F (2022). CB2 cannabinoid receptor expression is increased in 129S1/SvImJ mice: behavioral consequences. Front Pharm.

[64] Ortega-Alvaro A, Aracil-Fernández A, García-Gutiérrez MS, Navarrete F, Manzanares J (2011). Deletion of CB2 cannabinoid receptor induces schizophrenia-related behaviors in mice. Neuropsychopharmacology.

[65] Khella R, Short JL, Malone DT (2014). CB2 receptor agonism reverses MK-801-induced disruptions of prepulse inhibition in mice. Psychopharmacol (Berl).

[66] Cortez IL, Silva NR, Rodrigues NS, Pedrazzi JFC, Del Bel EA, Mechoulam R (2022). HU-910, a CB2 receptor agonist, reverses behavioral changes in pharmacological rodent models for schizophrenia. Prog Neuropsychopharmacol Biol Psychiatry.

[67] Siuciak JA, Chapin DS, McCarthy SA, Guanowsky V, Brown J, Chiang P (2007). CP-809,101, a selective 5-HT2C agonist, shows activity in animal models of antipsychotic activity. Neuropharmacology.

[68] Guo G, Tang J, Shi M, Yang C, Ou H, Chen W (2022). MK212, a 5-hydroxytryptamine 2C receptor agonist, reverses prepulse inhibition deficits in the medial prefrontal cortex and ventral hippocampus. Prog Neuropsychopharmacol Biol Psychiatry.

