# Supplementary material for: Adolescent exposure to the Spice/K2 cannabinoid JWH-018 impairs sensorimotor gating and alters cortical perineuronal nets in a sex-dependent manner
Source: Transl Psychiatry. 2023 May 25;13:176. doi: 10.1038/s41398-023-02469-4 (PMC10209055; doi:10.1038/s41398-023-02469-4)
Supplement: Supplementary file 1 — Supplemental Material [file 41398_2023_2469_MOESM1_ESM.pdf]

Figure S1

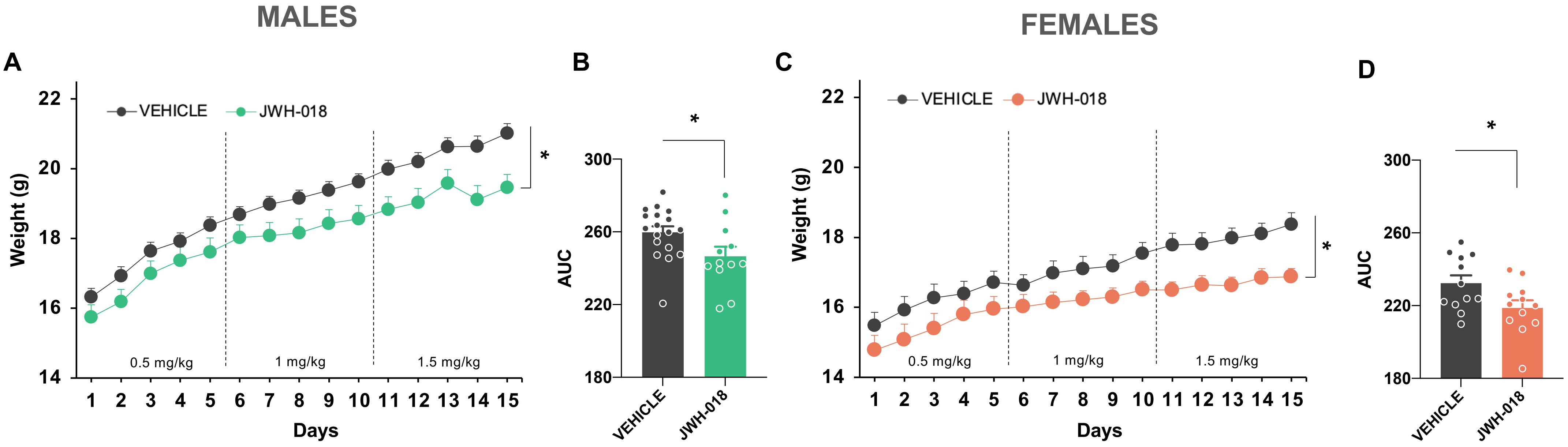

Fig. S1. Adolescent exposure to JWH-018 alters body weight in male and female mice. (A-D) Effects of JWH-018 treatment during the adolescence in body weight of a representative group of adolescent male (A,B) and female (C,D) mice (n= 12-18 mice per group). Daily weight in grams during the 15 days of the treatment with JWH-018 (A,C), and AUC values (B,D). Data are expressed as mean ± SEM. *p < 0.05 (comparison between JWH-018 and vehicle). AUC: area under the curve.

Figure S2

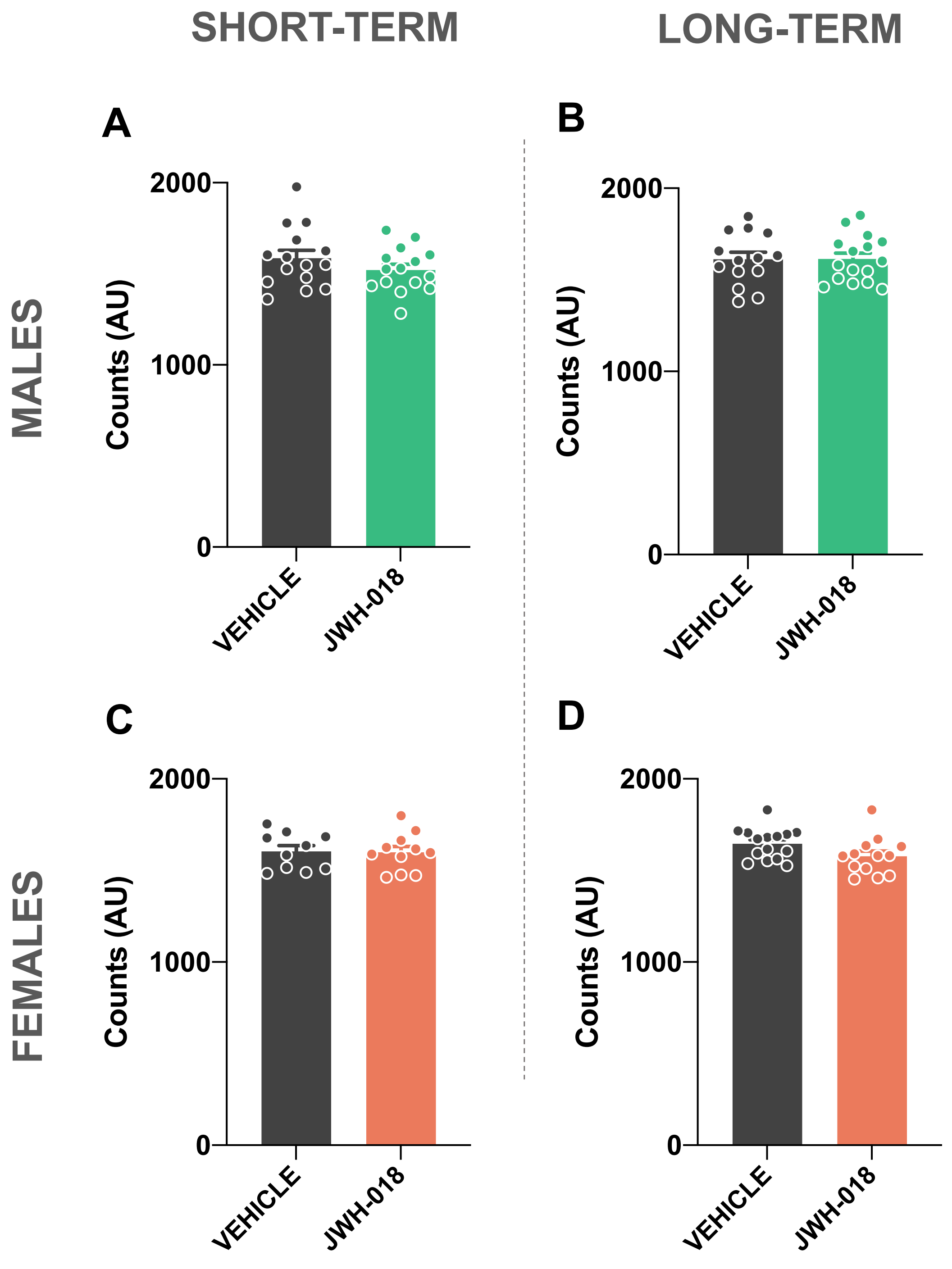

Fig. S2. Adolescent exposure to JWH-018 does not modify locomotion in male and female mice. (A-D) Effects of JWH-018 exposure during the adolescence on locomotor activity in male mice at short- (A) and long-term (B), and female mice at short- (C) and long-term (D) (n = 10-16 mice per group). Data are expressed as mean ± SEM. AU: arbitrary units.

Figure S3

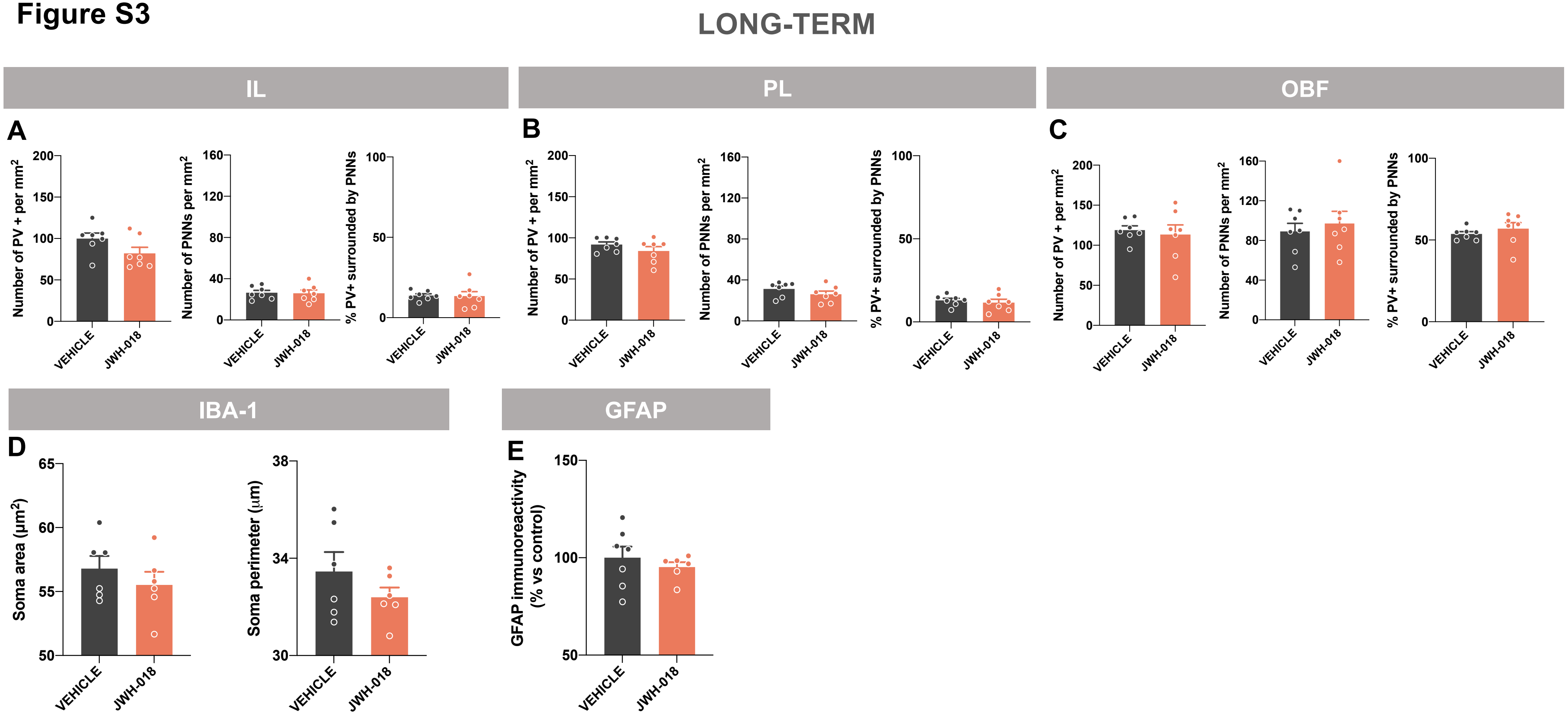

Fig. S3. Adolescent exposure to JWH-018 does not induce neurochemical alterations in female mice. (A-C) Effects JWH-018 exposure during the adolescence in the number of PV+, PNNs and PV+ surrounded by PNNs in the IL (A), PL (B), and OBF (C) of female mice at long-term (n = 7 mice per group). Tissue was obtained 24 h after the prepulse inhibition test. (D,E) Effects JWH-018 exposure during the adolescence in the area and perimeter of the soma of Iba-1-stained cells (D) and GFAP immunoreactivity (E) in the prefrontal cortex of female mice at long-term (n = 6-7 mice per group). Tissue was obtained 24 h after the prepulse inhibition test. Data are expressed as mean ± SEM. IL: infralimbic prefrontal cortex; PL: prelimbic prefrontal cortex; OBF: orbitofrontal cortex; PV+: positive parvalbumin neuron; PNNs: perineuronal nets. Iba-1; Ionized calcium-binding adapter molecule 1; GFAP: glial fibrillary acidic protein.

Figure S4

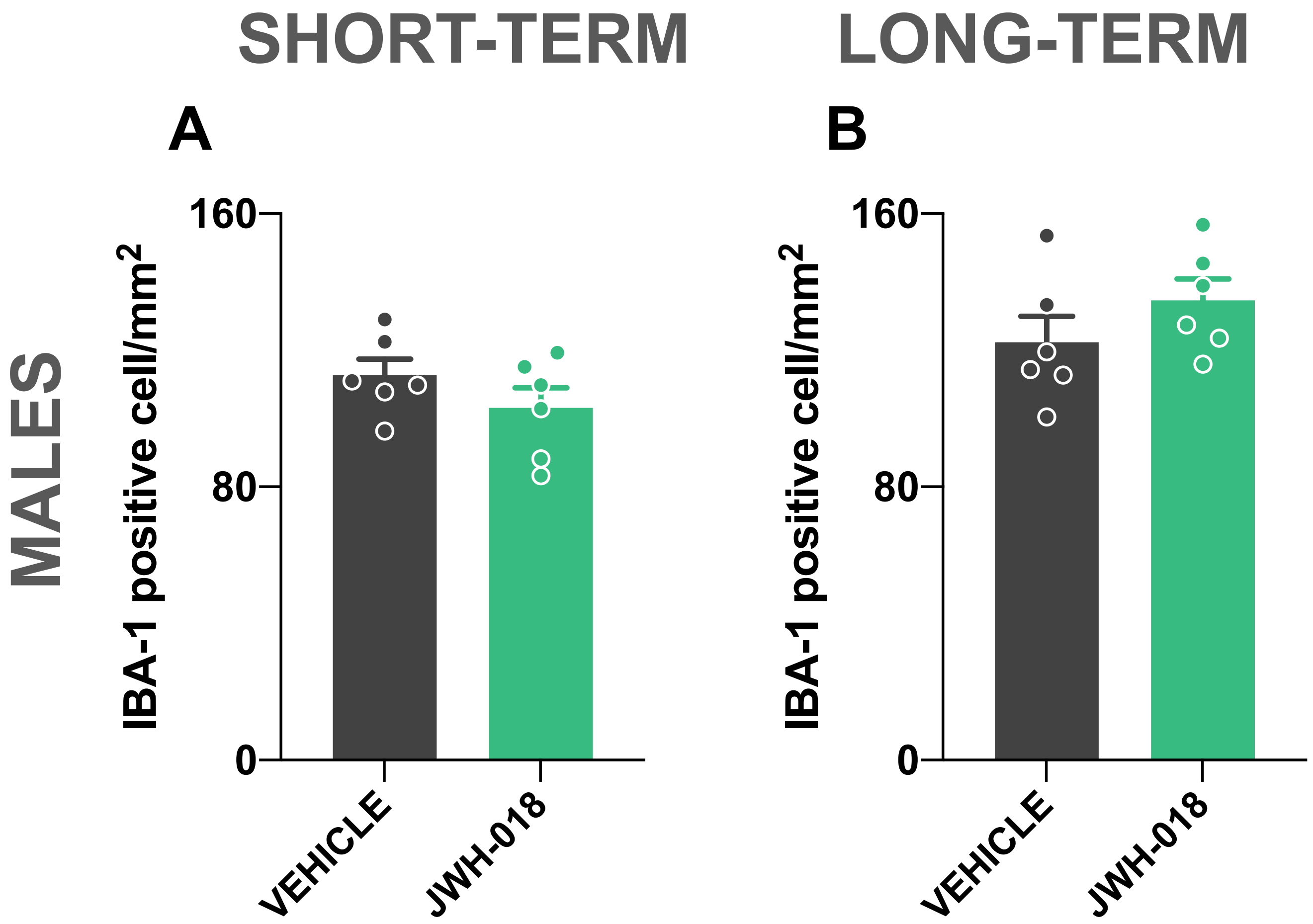

Fig. S4. Adolescent exposure to JWH-018 does not modify the number of Iba-1 + cells in male mice. (A-B) Effects JWH-018 exposure during the adolescence in the number of Iba-1+ in the prefrontal cortex of male mice at short- (A) and long-term (B). Tissue was obtained 24 h after the prepulse inhibition test. Data are expressed as mean ± SEM. Iba-1; Ionized calcium-binding adapter molecule 1.

Figure S5

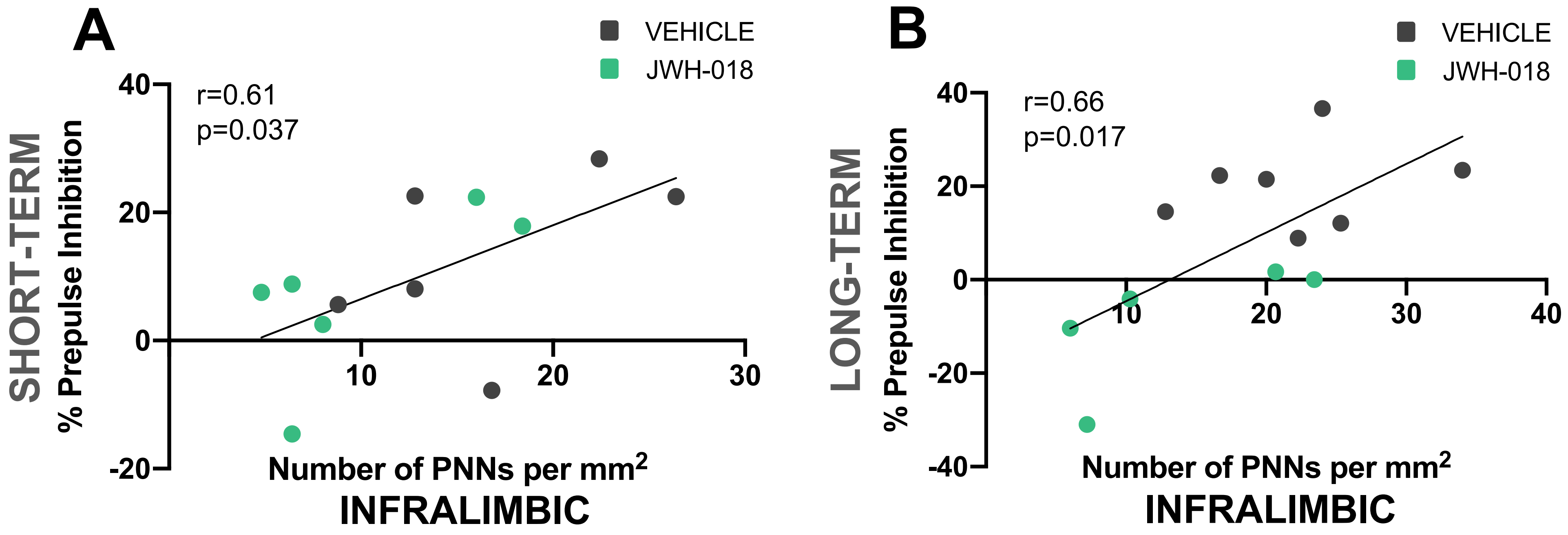

Fig. S5. Significant correlations between the percentage of prepulse inhibition and the density of PNNs in the IL in male mice. Adolescent exposure to JWH-018 leads to positive correlations between the percentage of prepulse inhibition (when representing the prepulses of 69 and 73 dB) and the density of PNNs in the IL in male mice at short- (A) and long-term (B). IL: infralimbic prefrontal cortex; PNNs: perineuronal nets; dB: decibels.
